# Supplementary material for: Mechanisms governing the pioneering and redistribution capabilities of the non-classical pioneer PU.1
Source: Nat Commun. 2020 Jan 21;11:402. doi: 10.1038/s41467-019-13960-2 (PMC6972792; doi:10.1038/s41467-019-13960-2)
Supplement: Supplementary file 7 — Source data [file 41467_2019_13960_MOESM7_ESM.zip › Source_Data/FigureS2/FigureS2B_MotifScanOutput/KmeansCluster10/homerResults/motif2.similar.html]

motif2

## Information for motif2

A
G
T
C
A
G
T
C
A
C
G
T
A
G
T
C
A
T
G
C
A
G
C
T
A
G
T
C
  
Reverse Opposite:  

A
C
T
G
C
T
G
A
A
T
C
G
A
C
T
G
C
G
T
A
C
T
A
G
A
C
T
G
  

|  |  |
| --- | --- |
| p-value: | 1e-334 |
| log p-value: | -7.700e+02 |
| Information Content per bp: | 1.901 |
| Number of Target Sequences with motif | 2385.0 |
| Percentage of Target Sequences with motif | 64.25% |
| Number of Background Sequences with motif | 13926.6 |
| Percentage of Background Sequences with motif | 31.51% |
| Average Position of motif in Targets | 103.0 +/- 53.5bp |
| Average Position of motif in Background | 101.2 +/- 71.1bp |
| Strand Bias (log2 ratio + to - strand density) | 0.0 |
| Multiplicity (# of sites on avg that occur together) | 1.46 |
| Motif File: | file (matrix) reverse opposite |

### Similar de novo motifs found

|  |  |  |  |  |  |  |  |
| --- | --- | --- | --- | --- | --- | --- | --- |
| Rank | Match Score | Redundant Motif | P-value | log P-value | % of Targets | % of Background | Motif file |
| 1 | 0.959 | A T G C G T A C G A C T A T G C A T G C A G C T G T A C A G C T | 1e-169 | -390.787381 | 31.25% | 12.79% | motif file (matrix) |
| 2 | 0.783 | C G T A A G T C G C A T G T A C G T A C A G T C A G C T A G T C A T G C A G C T A T G C | 1e-54 | -125.786383 | 7.49% | 2.33% | motif file (matrix) |
| 3 | 0.843 | T A C G C T G A T A G C T C A G T C G A C T A G C A T G C G T A C T A G T A C G C T G A | 1e-53 | -123.725469 | 20.93% | 11.56% | motif file (matrix) |
| 4 | 0.796 | G T A C G T C A A G T C G C A T G C A T G T A C G A T C A G C T T G A C G A T C G A C T A T G C | 1e-43 | -100.564694 | 4.31% | 1.01% | motif file (matrix) |
| 5 | 0.803 | T A C G C T G A T C A G T A C G C T G A C T A G T C A G C G T A C T A G A C T G C T G A T C A G | 1e-40 | -93.423776 | 8.89% | 3.72% | motif file (matrix) |
